# U. S. Veterinary Profession and the R.C.V.S.

**Published:** 1883-10

**Authors:** 


U. S. VETERINARY SURGEONS AND THE R. C.V. S.—We take pleasure in calling the attention of Veterinary Surgeons to the motion carried by Professor Walley at one of the meetings of the Council of the R. C.V. Surgeons admitting foreign veterinary surgeons into the college to study for a Diploma by attending only one winter and one summer session, and passing only in the subjects of the final examination.

Following are the extracts :

PROFESSOR WALLEY then moved "that the latter part of By-law 46 read thus :" A student holding a foreign or colonial veterinary diploma from any veterinary examining body recognized by the Council of the Royal College of Veterinary Surgeons shall be exempt from attendance on the course of lectures for the first two years and from the examinations at the end of those years respectively.

Professor Williams seconded and the President supported the motion, which was agreed to.

At a special meeting later Mr. DRAY proposed that the alteration in one of the By-laws, proposed by Prof. Walley at the last meeting and carried, be confirmed.

Mr. WRAGG seconded the motion.

GENERAL SIR FREDERIC FITZWYGRAM moved as an amendment, "That the alteration be not confirmed." He considered that it would be a step in the wrong direction altogether, Some years ago the Council compelled all students to attend a whole course of lectures and examinations. Since then he himself had proposed to relax that rule and to allow qualified men to prove that they were qualified, without attending the lectures. Any futher relaxation of the rule would, in his opinion, be injurious to the best interests of the college and to the qualification of the students.

The PRESIDENT said that, according to the unaltered by-law, any gentleman who was a graduate of a foreign or colonial school was admitted to the first and second year's examinations without attendance on lectures, the alteration would exempt them from all attendance on lectures and from the examinations at the end of those years. They would, however, have to study and to pass in Class 6. He (the President) supported the alteration, because he thought it unfair to a gentleman who had studied in a foreign school coming up in the M. B. subjects that he should be examined without having gone through the course of instruction. He had also in his mind that distinguished members of the profession holding foreign or colonial diplomas might come up for examination, and it would be very hard upon them to have to be examined in very elementary subjects.

The amendment not being seconded, the resolution was put and carried

